# Prevalence of vitamin D deficiency and the effect of vitamin D3 supplementation on response to anti-tuberculosis therapy in patients with extrapulmonary tuberculosis

**DOI:** 10.1186/s12879-024-09367-0

**Published:** 2024-07-09

**Authors:** Rasha Eletreby, Aisha Elsharkawy, Rahma Mohamed, Mai Hamed, Eman Kamal Ibrahim, Rabab Fouad

**Affiliations:** 1 Endemic Medicine Department, Faculty of Medicine, Cairo University, Cairo, Egypt; 2 Pulmonology Department, Faculty of Medicine, Cairo University, Cairo, Egypt; 3 Clinical and Chemical Pathology Department, Faculty of Medicine, Cairo University, Cairo, Egypt

**Keywords:** Extrapulmonary tuberculosis, Treatment outcome, Vitamin D deficiency

## Abstract

**Background:**

We aimed to assess serum 25-hydroxyvitamin D3 (25(OH)D3) concentrations in extrapulmonary tuberculosis (EPTB) patients and to evaluate the effect of vitamin D3 supplementation on their treatment course.

**Methods:**

Serum 25(OH)D3concentrations were measured in 47 newly diagnosed EPTB patients and 42 controls. Vitamin D-deficient EPTB patients were randomly assigned to receive 50,000 IU of vitamin D3 (cholecalciferol) orally once a week for 6 weeks (total 300,000 IU), followed by maintenance doses of 1000 IU a day besides anti-TB drugs or the first line anti-TB treatment only. Follow up serum 25(OH)D3 concentrations were measured after 3 months of starting vitamin D3 supplementation. Both groups were evaluated for clinical, laboratory, and radiological outcomes after treatment.

**Results:**

Serum 25(OH)D3 concentrations were significantly lower among TB cases (17.1 ± 5.5 nmol/L) compared to healthy controls (51.8 ± 27.3 nmol/L), and vitamin D deficiency was observed in all EPTB patients (*n* = 47). Patients in VD3 supplementation group had significantly higher weight gain and serum albumin level at 2 months and end of treatment, higher hemoglobin concentration at the end of treatment, significantly lower CRP and ESR at 2 months and at the end of treatment. In cases with TB pleurisy, a significant higher rate of full resolution of pleural fluid after 6 months of anti-TB treatment and shorter treatment duration were noted compared to the other group.

**Conclusions:**

Vitamin D deficiency is prevalent in EPTB patients, in whom, vitamin D supplementation is a useful adjunctive therapy to anti-TB drugs and improves treatment course.

## Introduction

Tuberculosis (TB) is a major public health threat and a leading cause of morbidity and mortality worldwide. The World health organization (WHO) estimates that there were 10.6 million incident cases of TB, and 1.6 million deaths due to TB in 2021 [1]. Egypt is ranked among the mid-level incidence countries. According to a WHO estimation of the TB burden in 2021, the incidence rate of TB cases was 10 cases per 100,000 inhabitants [2].

Several factors that could possibly affect the incidence and progression of TB have been identified; one of them is vitamin D deficiency [3].Vitamin D is an immunomodulatory micronutrient that affects both innate and adaptive immune responses [4]. Numerous studies have shown that vitamin D deficiency plays a role in TB prevalence, susceptibility to active disease and worse disease outcome in different populations [5, 6].

Two major forms of vitamin D, vitamin D3 (cholecalciferol) and vitamin D2 (ergocalciferol) are present naturally in small amounts in a few food sources, mainly oily fish for vitamin D3 and mushrooms and egg yolks for vitamin D2 [7].

D2 and D3 are structurally similar, but D2 has an extra methyl group and double bonds. However, these structural differences do not affect the metabolic activation of vitamin D; therefore, both forms are considered equivalent [8]. Vitamin D2 is most commonly added to foods given the paucity of naturally occurring VD-rich foods, whereas vitamin D3 is mainly synthesized in the skin during exposure to ultra violet B (UV-B) radiation from 7-dehydrocholesterol, but also can be obtained from dietary intake [9]. Subsequently, two hydroxylation reactions occur in the liver and kidney to form 25-hydroxyvitamin D3 and the active hormone form of vitamin D, 1,25-dihydroxyvitamin D3 (calcitriol) by essential enzymes 25- and 1-α-hydroxylases, respectively [9]. 1-α-hydroxylase is predominantly found in the proximal tubular cells of the kidney but has also been described in many other cell types including skin, brain, prostate, pancreas and macrophages [10].

In 2006, Liu and colleagues showed that the triggering of toll-like receptors (TLRs) on the cell surface of human macrophages by tuberculosis protein caused the upregulation of genes leading to the production of the vitamin D receptor (VDR), a polymorphic nuclear receptor that regulates the expression of genes crucial for immune function and involved in cytokine production and the vitamin D-1-α-hydroxylase enzyme, leading to both increased levels of active vitamin D and increased potential binding of calcitriol with the VDR [11]. Calcitriol is then induces the production of several endogenous antimicrobial peptides, particularly cathelicidin LL-37 and defensin beta2 [11]. Additionally, it induces oxidative species, including NO and H2O2 [12] and suppresses matrix metalloproteinase enzymes linked to the pathogenesis of pulmonary cavitation [13]. The resulting increased cathelicidin production, which is totally dependent on vitamin D to be produced, leads to killing of intracellular mycobacterium tuberculosis [14].

In the pre-antibiotic era, cod liver oil, UVB phototherapy, sunshine, oral vitamin D, and injectable vitamin D were all shown to be able to safely treat tuberculosis [15]. The Nobel prize in medicine in 1903 was awarded to Dr. Neils Ryberg Finsen acknowledging his success in curing hundreds of long-standing cases of lupus vulgaris (cutaneous tuberculosis infections) with refracted light rays from an electric arc lamp, and this therapeutic approach became the standard of care for treating TB until the discovery of antibiotics in the 1940’s [15]. Furthermore, several reports from the 1940s document cases where patients with lupus vulgaris were safely and effectively cured by taking daily doses of 100,000 IU to 150,000 IU of oral vitamin D2 as the sole treatment for 2 to 3 months, all without developing complications related to hypercalcemia or withdrawing from therapy [16–19].

However, randomized clinical trials of vitamin D supplementation in patients with pulmonary TB performed over the past few decades have produced mixed findings on sputum culture/smear conversion and other TB treatment outcomes [20–28]. In addition, to the best of our knowledge; no randomized trials have examined the effect of vitamin D supplementation on the treatment of extrapulmonary TB (EPTB). We conducted the present study to assess serum 25-hydroxyvitamin D3 (25(OH)D3) concentrations in newly diagnosed patients with EPTB, its relation to disease activity and worse disease outcome and the effect of vitamin D3 supplementation on response to anti-TB treatment in EPTB patients.

## Materials and methods

### Study design and participants

A total of 47 EPTB patients who presented to the TB outpatient clinic, at Cairo University Hospital, Egypt were consecutively recruited over the period from November 2020 till September 2021 and followed up for 6–12 months after starting anti-TB treatment. The study was designed in two phases: the first phase was a case-control study aiming at assessing serum 25(OH)D3 concentrations in newly diagnosed EPTB patients compared with healthy controls, and the second phase was a pilot randomized controlled clinical trial to evaluate the role of combined vitamin D3 supplementation to standard TB therapy in the treatment course of EPTB patients compared to standard therapy alone.

Inclusion criteria were: [1] adult patients (age ≥ 18 years old); [2] Naïve for anti-TB treatment. As per WHO guidelines [29, 30], the diagnosis of EPTB cases was confirmed according to the following: (a) Microbiological evidence in form of acid-fast bacilli (AFB) positivity on tissue or fluid staining, growth of AFB on culture of tissue or fluid or positive GeneXpert MTB/RIF testing on tissue or fluid sample; (b) Histological findings of epithelioid cell granulomas with caseous necrosis in tissue biopsies. Healthy individuals who did not have any history of TB or contacts with TB patients and had no symptoms or signs of TB or pathologic findings in radiology were included in control group. These volunteers were matched to patients by age (± 5years),  gender and season (± 1 month). The exclusion criteria were subjects with a history of diabetes mellitus (DM), hepatic disease, renal failure, malignancy, human immunodeficiency virus (HIV) infection, multi-drug resistant TB (MDR-TB), pregnancy and lactation, sarcoidosis, hyperparathyroidism or those taking vitamin D supplementation in the previous 12 months, medications that affect vitamin D levels, corticosteroids, or immunosuppressive agents. This study was approved by the Research Ethics Committee, Faculty of Medicine, Cairo University (Approval code: MD-241-2020).Written informed consent was taken from all the participants.

### Study procedure

#### Phase I

In phase I, all the participants were subjected to detailed history taking including demographic data, comorbidities, concomitant medications, smoking, intravenous (IV) drug use, and alcohol intake. EPTB cases were also assessed for TB associated constitutional symptoms (i.e. fever, night sweats, loss of weight, loss of appetite, fatigue) and local symptoms (e.g. cough, shortness of breath, chest pain, enlarged peripheral lymph node, abdominal pain, diarrhea, bleeding per rectum, abdominal distention …etc ). Height and weight were measured, and body mass index (BMI) was calculated using the formula: weight (kg)/height (m2).

Laboratory evaluation included complete blood count (CBC), liver biochemical profile (total serum bilirubin, liver enzymes [alanine aminotransferase (ALT), aspartate aminotransferase (AST)], and serum albumin), serum creatinine, and serum calcium. For cases, baseline inflammatory markers (Erythrocyte sedimentation rate (ESR) and C-reactive protein (CRP)) were also measured. Albumin-adjusted serum calcium concentrations were calculated using the following equation: calcium (mg/dL) = total calcium (mg/dL) + 0.8 × [4 - albumin (g/dL)]. Hypocalcemia, normo-calcemia or hypercalcemia were defined when corrected serum calcium levels were below 8.5 mg/dl, 8.5–10.10 mg/dl or exceeding 10.10 mg/dl, respectively [31]. All chemistry analysis was performed on Beckman Coulter AU680 autoanalyzer according to the manufacturer’s methods.

##### Measurement of serum 25(OH)D3 concentration

We measured serum concentrations of 25(OH)D3 for cases, before starting anti- TB therapy, and for controls using High Performance Liquid Chromatography (HPLC-UV method). It has sensitivity up to: 1.5 ng/mL with a linearity: 2–500 ng/mL for detection. To convert ng/mL to nmol/L, multiply by 2.496. Serum 25(OH)D3 concentrations < 30 nmol/L were defined as vitamin D deficiency, 25(OH)D3 concentrations from 30 to 50 nmol/L were considered as insufficient, and serum 25(OH)D3 concentrations > 50 nmol/L were considered normal [32].

All EPTB patients underwent radiological investigations according to TB site including: (a) abdominal ultrasonography to examine amount of ascites, septations or loculation, peritoneal thickening, thickened omentum, mesenteric thickening, intestinal involvement, any focal lesion or enlarged abdominal lymph nodes; (b) Lymph node (LN) sonography to assess location, size, number, shape, and echogenicity of enlarged LNs, formation of fistulae and/or abscesses, as well as any change in the size of nodes during anti-TB treatment; (c) Computed tomography (CT) in TB pleurisy cases to assess the amount of pleural effusion, pleural thickening, and LN enlargement.

#### Phase II

In phase II, EPTB patients with concomitant Vitamin D deficiency were randomly assigned into two groups: Group I (“**Vitamin D**” group) included patients who received both vitamin D supplementation and the first line anti-tuberculous treatment. Vitamin D supplementation was given according to the recommended dose [33] i.e. 50,000 IU vitamin D3 (cholecalciferol) orally once a week for 6 weeks (total 300,000 IU), followed by maintenance doses of 1000 IU a day. The first line anti-TB treatment included [ Isoniazide (5 mg/kg /day PO), Rifampin (10 mg/kg/day PO), Ethambutol (15 mg/kg/day PO) and Pyrazinamide(15–30 mg/kg/day PO)] for 2 months then Isonizide and Rifampin for 4–10 months; while Group II (“**No vitamin D**”) received the first line anti-TB treatment only. Follow up serum 25(OH)D3 concentrations were measured after 3 months of starting vitamin D3 supplementation. Participants were randomized using identical sealed envelopes technique.

#### Outcome measures

Response to anti-TB treatment was compared between the two studied groups at two time points; after the intensive phase of anti-TB treatment at 2 months and at the end of treatment, according to the following parameters;

i. Improvement of (constitutional and local) symptoms. The number of symptoms the participants reported at baseline and symptom count ratio (SCR) derived by dividing the number of symptoms reported as much better or resolved at the follow-up visit by the total number of symptoms reported at baseline.
ii. Changes in weight: patients were weighed at each follow-up visit, and weight change was recorded in kilograms (kg) and as percentage change in weight at 2 months and end of treatment compared to baseline.
iii. Follow up laboratory investigations including; CBC, liver biochemical profile (AST, ALT, and serum albumin), inflammatory markers (ESR and CRP).
iv. Change in serum 25(OH)D3 concentrations (in the patient group who received vitamin D3 supplementation besides the first-line anti-TB drugs) three months after starting vitamin D3 supplementation.
v. Follow up radiological investigations repeated at 2 months, 6months and at the end of treatment to evaluate regression of lymph nodes size by LN ultrasound, amount pleural effusion by CT chest and regression of amount peritoneal effusion by abdominal ultrasound.

#### Adverse effects

Patients who received vitamin D3 supplement were questioned for the following adverse effects related to hypercalcemia e.g. anorexia, nausea, vomiting, excessive thirst, symptoms of kidney stones, and confusion. Serum levels of calcium were measured at the end of 2nd month.

### Statistical methods

Data were statistically described in terms of mean ± standard deviation (± SD), median and range, or frequencies and percentages when appropriate. Numerical data were tested for the normal assumption using Kolmogorov Smirnov test. Comparison of numerical variables between the study groups was done using Student *t* test for independent samples in comparing normally distributed data and Mann Whitney *U* test for independent samples for comparing non-normal data. Over time comparison of numerical variables was done using paired *t* test for normally distributed data and Wilcoxon signed rank test for paired (matched) samples for non-normal data. For comparing categorical data, Chi-square (χ^2^) test was performed. Exact test was used instead when the expected frequency is less than 5. Over time categorical data were compared using McNemar test. Two-sided *p* values less than 0.05 was considered statistically significant. IBM SPSS (Statistical Package for the Social Science; IBM Corp, Armonk, NY, USA) release 22 for Microsoft Windows was used for all statistical analyses.

## Results

This study included 47 EPTB patients and 42 healthy controls. Table 1 shows the baseline characteristics and laboratory data of study participants. Mean age for TB cases was 32.0 ± 10.6 years, 53.2% were males. Compared to controls, EPTB patients were more often unemployed (*P* = 0.011) and had significantly lower BMI (*p* < 0.001), significantly lower hemoglobin concentration and lower serum albumin levels (*P* = 0.023 and P = < 0.001, respectively), significantly lower serum 25(OH)D3 concentrations (17.1 ± 5.5 nmol/L) compared to healthy controls (51.8 ± 27.3 nmol/L, *p* < 0.001). Vitamin D deficiency was observed in all EPTB cases (*n* = 47, 100.0%), while in the control group, only 9.5% had vitamin D deficiency, and 59.5% had vitamin D insufficiency. Table 2 shows the distribution of extrapulmonary TB among TB cases (*n* = 47); 38.3% had TB pleurisy, 34% had TB lymphadenitis, 17% had TB peritonitis and 5 patients (10.6%) had both TB peritonitis and pleurisy.

**Table 1:** Baseline characteristics and laboratory data of the TB cases and healthy control groups

Parameters			TB cases (*n* = 47)	Healthy controls (*n* = 42)	*P* value
Age		Mean ± SD	32.0 ± 10.6	31.9 ± 10.7	0.968
		Range	18–57	18–57	
Gender, n (%)		Female	22(46.8)	20(47.6)	0.939
		Male	25(53.2)	22(52.4)	
BMI (kg/m2)		Mean ± SD	20.5 ± 3	25.3 ± 3.1	**< 0.001**
BMI (categorized), n (%)		< 18.5 (underweight)	11(23.4)	2(4.8)	**< 0.001**
		18.5–24.9( Normal)	33(70.2)	18(42.9)	
		25.0-29.9(Overweight) and ≥ 30.0 (Obese)	3(6.4)	22(52.3)	
Educational Level, n (%)		Primary Education and below	16(34.0)	14(33.3)	0.934
		Secondary Education	11(23.4)	8(19.0)	
		Technical Education	11(23.4)	10(21.4)	
		Higher Education	9(19.1)	10(23.8)	
Employment status, n (%)		Employed	21(44.7)	30(71.4)	**0.011**
		Unemployed	26(55.3)	12(28.6)	
Residence, n (%)		Rural	9(19.1)	6(14.3)	0.541
		Urban	38(80.9)	36(85.7)	
Special habits and risk factors for TB:					
Smoking, n (%)		Yes	17(36.2)	10(23.8)	0.205
IV Drug use, n (%)		Yes	4(8.5)	0	0.119
Alcohol use, n (%)		Yes	4(8.5)	0	0.119
Laboratory parameters, Mean ± SD					
Hb (g/dl)			10.6 ± 1.2	17.1 ± 19.3	**0.023**
TLC x10^3 /mm3			6.3 ± 2.3	7.6 ± 9.5	0.361
PLTx10^3 /mm3			247.2 ± 56.9	232.9 ± 50.4	0.261
Total bilirubin (mg/dl)			0.9 ± 0.2	0.8 ± 0.3	**0.022**
AST (IU/L)			36.1 ± 5.1	36.3 ± 14.3	0.904
ALT(IU/L)			37.5 ± 13.8	34.8 ± 15.4	0.392
Serum creatinine (mg/dl)			0.8 ± 0.2	0.8 ± 0.2	0.785
Albumin (g/dl)			3.6 ± 0.3	4.3 ± 0.3	**< 0.001**
Calcium (mg/dl)			9.2 ± 0.3	9.1 ± 0.3	0.271
Vitamin D level (nmol/L)			17.1 ± 5.5	51.8 ± 27.3	**< 0.001**
Baseline vitamin D status, n (%)					
Vitamin D deficiency			47(100.0)	4(9.5)	**< 0.001**
Vitamin D insufficiency			0	25(59.5)	
Vitamin D sufficiency			0	13(31.0)	
Season of sampling, n (%)					
Autumn (Sep-Nov)			7(14.9)	7(16.7)	0.993
Spring (Mar-May)			11(23.4)	9(21.4)	
Summer (Jun-Aug)			22(46.8)	20(47.6)	
Winter (Dec-Feb)			7(14.9)	6(14.3)	

BMI; body mass index; Hb: Hemoglobin; TLC: Total leucocyte Count; PLT: Platelet count; AST: Aspartate aminotransferase; ALT: Alanine aminotransferase

*P-value is significant if ≤ 0.05

**Table 2:** Distribution of extrapulmonary TB patients according to TB site and treatment completion

Parameters			Total TB cases (*n* = 47)	“Vitamin D Supplement” group (*n* = 23)	“No vitamin D Supplement” group (*n* = 24)	*P* value
**TB site**	TB lymphadenitis		16(34.0)	8(34.8)	8(33.3)	0.878
	TB Peritonitis		8(17.0)	3(13.0)	5(20.8)	
	TB Peritonitis and Pleurisy		5(10.6)	3(13.0)	2(8.3)	
	Tuberculous Pleurisy		18(38.3)	9(39.1)	9(37.5)	
**Treatment completion**		Died	1(2.1)	0	1(4.2)	0.613
		Lost-to-follow up	4(8.5)	2(8.7)	2(8.3)	
		Treatment completed	42(89.4)	21(91.3)	21(87.5)	

In phase II of the study, EPTB patients were randomly assigned to either the “Vitamin D” group or the “No vitamin D” group. Both groups had similar baseline characteristics. Out of the 47 TB cases, 1 patient (2.1%) died and 4 (8.5%) were lost-to-follow-up before first follow-up visit. Therefore, a total of 42 EPTB patients were followed during treatment, 21 patients in each group **(**Table 2**).**

The main reported symptoms at baseline (*n* = 42) were fatigue (100%), fever (95.2%), weight loss (92.9%), night sweats (73.8%), loss of appetite (69%), and local symptoms related to the site of TB infection such as peripheral lymph node swelling (14/42,33.3%), abdominal pain (12/42, 28.6), abdominal distension (12/42, 28.6%),chest pain (5/42,11.9%), dyspnea(17/42, 40.5%),and cough(6/42, 14.3%) **(**Table 3**).**

**Table 3:** Presenting constitutional and local TB symptoms at baseline, 2 months and at the end of anti-TB treatment (EOT)

Parameters		Total TB case (*n* = 42)	“Vitamin D supplement” group (*n* = 21)	“No vitamin D supplement” group (*n* = 21)	*P* value
Fever, n (%)	Baseline	40(95.2)	20(95.2)	20(95.2)	1.000
	2months	3(7.1)	2(9.5)	1(4.8)	0.549
	EOT	0(0.0)	0(0.0)	0(0.0)	-
Weight loss, n (%)	Baseline	39(92.9)	19(90.5)	20(95.2)	0.549
	2months	1(2.4)	0(0.0)	1(4.8)	0.311
	EOT	0(0.0)	0(0.0)	0(0.0)	-
Night sweats, n (%)	Baseline	31(73.8)	16(76.2)	15(71.4)	0.726
	2months	2(4.8)	1(4.8)	1(4.8)	1.000
	EOT	0(0.0)	0(0.0)	0(0.0)	-
Loss of appetite, n (%)	Baseline	29(69.0)	15(71.4)	14(66.7)	0.739
	2months	3(7.1)	2(9.5)	1(4.8)	0.549
	EOT	0(0.0)	0(0.0)	0(0.0)	-
Fatigue, n (%)	Baseline	42(100)	21(100)	21(100)	-
	2months	8(19.0)	3(14.3)	5(23.8)	0.432
	EOT	0(0.0)	0(0.0)	0(0.0)	-
Enlarged peripheral LN, n (%)	Baseline	14(33.3)	7(33.3)	7(33.3)	1.000
	2months	12(28.6)	6(28.6)	6(28.6)	1.000
	EOT	0(0.0)	0(0.0)	0(0.0)	-
Painful LN (*n* = 14)	Baseline	6(42.9)	4(57.1)	2(28.6)	0.280
	2 months	2(14.3)	1(14.3)	1(14.3)	1.000
	EOT	0(0.0)	0(0.0)	0(0.0)	-
LN discharge/fistula (*n* = 14)	Baseline	2(14.3)	2(28.6)	0(0.0)	0.127
	2 months	0(0.0)	0(0.0)	0(0.0)	-
	EOT	0(0.0)	0(0.0)	0(0.0)	-
Abdominal pain, n (%)	Baseline	12(28.6)	6(28.6)	6(28.6)	1.000
	2months	3(7.1)	2(9.5)	1(4.8)	0.549
	EOT	0(0.0)	0(0.0)	0(0.0)	-
Abdominal distention, n (%)	Baseline	12(28.6)	6(28.6)	6(28.6)	1.000
	2months	6(14.3)	2(9.5)	4(19.0)	0.378
	EOT	0(0.0)	0(0.0)	0(0.0)	-
Chest pain, n (%)	Baseline	5(11.9)	3(14.3)	2(9.5)	0.634
	2months	0(0.0)	0(0.0)	0(0.0)	-
	EOT	0(0.0)	0(0.0)	0(0.0)	-
Dyspnea, n (%)	Baseline	17(40.5)	9(42.9)	8(38.1)	0.753
	2months	5(11.9)	2(9.5)	3(14.3)	0.634
	EOT	0(0.0)	0(0.0)	0(0.0)	-
Cough, n (%)	Baseline	6(14.3)	4(19.0)	2(9.5)	0.378
	2months	0(0.0)	0(0.0)	0(0.0)	-
	EOT	0(0.0)	0(0.0)	0(0.0)	-

All 42 patients showed improvement in presenting symptoms after two months of treatment and complete resolution at the end of the anti-TB therapy, with no significant difference between the two groups regarding the number of symptoms reported at baseline, number of improved symptoms at 2 months and at the end of treatment (*p* = 0.282, 0.254 and 0.282) as well as symptom count ratio (SCR) at 2 months and at the end of treatment. Moreover, there was no significant difference in the mean treatment duration between both groups (*p* = 0.065) except in subgroup of patients with TB pleurisy, in which the patients in VD3 supplementation group had shorter treatment duration (*p* = 0.021) **(**Table 4**)**.

**Table 4:** Improvement of presenting (constitutional and local) symptoms at 2 months and at the end of anti-TB treatment, and treatment duration for each TB site

Parameters		Total TB case (*n* = 42)	TB cases		*P* value
			“Vitamin D supplement” group **(*n*** **= 21)**	“No vitamin D supplement” group **(*n*** **= 21)**	
Number of symptoms at baseline, Mean ± SD		6.1 ± 1.3	6.3 ± 1.4	5.9 ± 1.2	0.282
Number of improved symptoms	2 months	5 ± 1.5	5.3 ± 1.6	4.8 ± 1.3	0.254
	EOT	6.1 ± 1.3	6.3 ± 1.4	5.9 ± 1.2	0.282
Symptom count ratio (SCR)	2 months	0.8 ± 0.2	0.8 ± 0.2	0.8 ± 0.2	0.696
	EOT	1 ± 0.0	1 ± 0.0	1 ± 0.0	-
Treatment duration, Mean ± SD		7.8 ± 1.8	7.3 ± 1.5	8.3 ± 1.9	0.065
Treatment duration for each TB site, n (%)					
TB lymphadenitis	6months	7(50.0)	4(57.1)	3(42.9)	0.593
	9months	7(50.0)	3(42.9)	4(57.1)	
TB Peritonitis +/- Pleurisy	9months	10(83.3)	6(100.0)	4(66.7)	0.121
	12months	2(16.7)	0	2(33.3)	
TB Pleurisy	6months	12(75.0)	8(100)	4(50.0)	0.021
	9months	4(25.0)	0	4(50.0)	

**Fig. 1:**
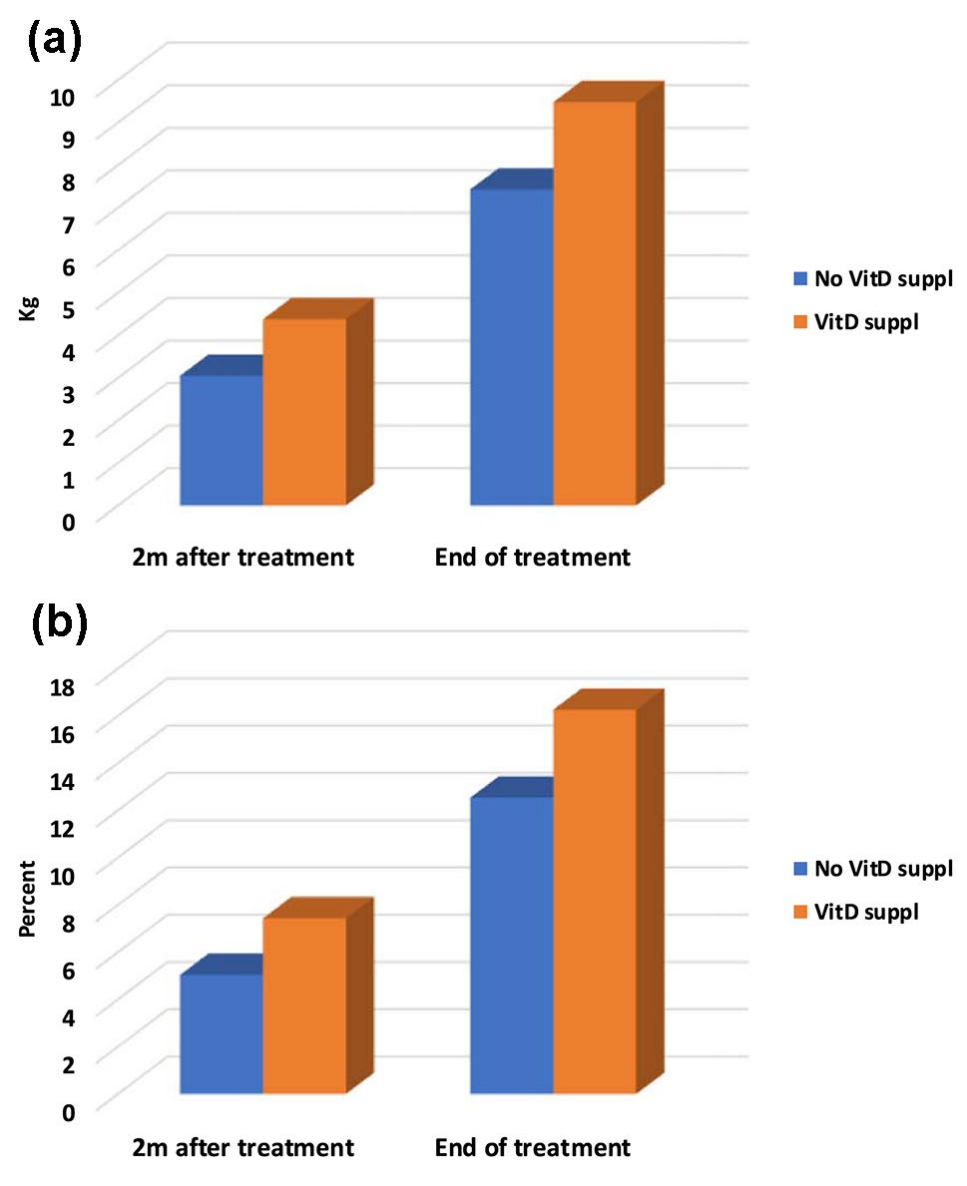
(**a**) Mean weight increase (kg) at 2 months and at the end of treatment between vitamin D supplementation and no supplementation groups. (**b**) Mean %weight increase (kg) at 2 months and at the end of treatment between vitamin D supplementation and no supplementation groups

An overall significant weight gain was observed in all TB cases at 2 months and at treatment completion compared to baseline, with patients in VD3 supplementation group had significantly higher mean weight gain (4.4 ± 1.8 kg vs. 3.1 ± 1 kg at 2 months (*p* = 0.004) and 9.5 ± 3 kg vs. 7.4 ± 1.7 kg at the end of TB treatment (*p* = 0.010)) and percentage of weight gain (7.4 ± 2.9%vs. 5 ± 1.6% at 2 months(*p* = 0.002) and 16.3 ± 6%vs. 12.5 ± 4% at the end of treatment(*p* = 0.023) (Fig. 1a& b).

As shown in Table 5, patients in VD3 supplementation group had significantly higher hemoglobin concentration at the end of treatment (*p* < 0.001),higher serum albumin level at 2 months and at the end of treatment(*p* = 0.007 and *P* < 0.001), lower CRP level (*p* = 0.002 and *P* < 0.001) and lower ESR at 2 months and at the end of treatment(*p* = 0.02 and *P* < 0.001). Additionally, patients in VD3 supplementation group had a significant increase in serum 25(OH)D3 concentrations after 3 months of supplementation (71.6 ± 7.1nmol/L)compared to baseline (17.3 ± 5.6nmol/L, *p* < 0.001). None of the patients experienced hypercalcemia or any adverse events related to anti-tuberculosis treatment.

**Table 5:** Changes in laboratory parameters in the studied groups at 2 months after starting anti-TB treatment and at the end of treatment (EOT)

Parameters		Total (*n* = 42)	“Vitamin D Supplement” group (*n* = 21)	“No vitamin D Supplement” group (*n* = 21)	P value
		Mean ± SD	Mean ± SD	Mean ± SD	
Hb (g/dl)	Baseline	10.5 ± 1.2	10.4 ± 1.1	10.6 ± 1.3	0.637
	2 months	12.2 ± 1	12.5 ± 1	11.9 ± 1	0.091
	EOT	13.6 ± 0.8	14.1 ± 0.5	13.1 ± 0.6	**< 0.001**
	**P-value**	< 0.001* < 0.001 **	< 0.001* < 0.001 **	< 0.001* < 0.001**	
TLC x10^3 /mm3	Baseline	6.2 ± 2.2	5.9 ± 2.1	6.6 ± 2.2	0.257
	2 months	6.2 ± 2	5.8 ± 1.8	6.7 ± 2.2	0.150
	EOT	6.1 ± 2.2	5.9 ± 2.4	6.3 ± 2.1	0.570
	**P-value**	0.971* 0.782**	0.901* 0.915**	0.945* 0.568 **	
PLTx10^3 /mm3	Baseline	250.5 ± 56	248.1 ± 58.7	252.9 ± 54.5	0.787
	2 months	249.8 ± 50	248.4 ± 47.4	251.1 ± 52.8	0.864
	EOT	252.4 ± 40.4	251.2 ± 36.7	253.7 ± 44.6	0.845
	**P-value**	0.946* 0.858**	0.985* 0.838**	0.915* 0.961**	
ALT (IU/L)	Baseline	37.6 ± 14.2	37.5 ± 16	37.6 ± 12.5	0.983
	2 months	43 ± 11.3	43 ± 10.1	42.9 ± 12.6	0.957
	EOT	37.7 ± 9.2	37.3 ± 11.8	38.1 ± 5.8	0.805
	**P-value**	0.068* 0.969**	0.189* 0.972**	0.224* 0.888**	
AST (IU/L)	Baseline	36.3 ± 5.2	37 ± 5.3	35.7 ± 5.1	0.411
	2 months	41.5 ± 8.1	40.1 ± 8.3	43 ± 7.9	0.260
	EOT	36 ± 5	35.5 ± 4.3	36.5 ± 5.7	0.525
	**P-value**	0.001* 0.785**	0.092* 0.341**	0.005* 0.611**	
Total bilirubin (mg/dl)	Baseline	0.9 ± 0.2	1 ± 0.3	0.9 ± 0.2	0.579
	2 months	1 ± 0.2	1 ± 0.2	1 ± 0.3	0.363
	EOT	1 ± 0.3	0.9 ± 0.3	1 ± 0.3	0.399
	**P-value**	0.215* 0.541**	0.260* 0.729**	0.516* 0.301**	
Albumin (g/dL)	Baseline	3.6 ± 0.3	3.5 ± 0.4	3.6 ± 0.3	0.568
	2 months	4.2 ± 0.3	4.3 ± 0.3	4.1 ± 0.3	**0.007**
	EOT	4.4 ± 0.3	4.6 ± 0.3	4.2 ± 0.2	**< 0.001**
	**P-value**	< 0.001* < 0.001 **	< 0.001* < 0.001 **	< 0.001* < 0.001 **	
ESR	Baseline	63.3 ± 15.6	63.6 ± 12.4	63 ± 18.7	0.900
	2 months	29.3 ± 8.3	26.3 ± 4.9	32.2 ± 9.9	**0.020**
	EOT	11.2 ± 2	10 ± 1.5	12.4 ± 1.6	**< 0.001**
	**P-value**	< 0.001* < 0.001 **	< 0.001* < 0.001 **	< 0.001* < 0.001 **	
CRP	Baseline	55.3 ± 13.9	54.8 ± 12.5	55.9 ± 15.5	0.802
	2 months	25.5 ± 6.9	22.3 ± 5.4	28.7 ± 6.8	**0.002**
	EOT	5.1 ± 3.1	3 ± 1.7	7.2 ± 2.7	**< 0.001**
	**P-value**	< 0.001* < 0.001 **	< 0.001* < 0.001 **	< 0.001* < 0.001 **	
Calcium (mg/l)	Baseline	9.2 ± 0.4	9.1 ± 0.4	9.2 ± 0.3	0.738
	2 months	9.2 ± 0.3	9.2 ± 0.4	9.2 ± 0.3	0.762
	**P-value**	0.904	0.736^*^	0.758^*^	
Vitamin D level (nmol/L)	Baseline	17.2 ± 5.7	17.3 ± 5.6	17 ± 5.8	0.861
	3 months	**-**	71.6 ± 7.1	**-**	-
	**P-value**	**-**	**< 0.001**	**-**	-

CRP: C-reactive protein, ESR: erythrocyte sedimentation rate

*P-value between Baseline and 2 months. ** P-value between Baseline and EOT

As shown in Table 6, all cases with TB lymphadenitis demonstrated a significant regression in lymph node size at the end of anti-TB treatment compared to the baseline (p < 0.001), with no significant differences between the “Vitamin D” and the “No vitamin D” groups. Likewise, regression in amount of ascites was detected in 75% of cases with TB peritonitis (n = 9/12) at 2 months and in all cases after 6 months of anti-TB treatment, however, with no complete resolution of ascites. So, treatment was extended for additional 3 months in 10 patients (83.3%) and for additional 6 months in 2 patients (16.7%), with no significant differences between the “Vitamin D” and the “No vitamin D” groups. In cases with TB pleurisy, regression of pleural effusion was noted in all patients at 2 months of anti-TB therapy, and full resolution of effusion occurred in 14.3% and 81% of patients at 2 months and 6 months, respectively, with higher rate of resolution of pleural fluid at 6 months in the “Vitamin D” group (100% vs. 60%, p = 0.020).

**Table 6:** Changes in radiological findings according to TB site

Parameters		Total TB cases	“Vitamin D Supplement” group	“No vitamin D Supplement” group	*P* value
**LN ultrasound findings in TB lymphadenitis cases** (***n*** **= 14)**					
Enlarged LN site, n (%)	axillary	4(28.6)	2(28.6)	2(28.6)	0.370
	Cervical	7(50.0)	4(57.1)	3(49.2)	
	Cervical and axillary	2(14.3)	0(0.0)	2(28.6)	
	Cervical and inguinal	1(7.1)	1(14.3)	0(0.0)	
LN site, n (%)	Bilateral	2(14.3)	0(0.0)	2(28.6)	0.127
	Unilateral	12(85.7)	7(100.0)	5(71.4)	
Matted nodes, n (%)	Yes	8(57.1)	4(57.1)	4(57.1)	1.000
Discharge /fistula, n (%)	Yes	2(14.3)	2(28.6)	0(0.0)	0.127
Largest LN size /cm	Baseline	4.3 ± 0.7	4.4 ± 0.8	4.1 ± 0.6	0.401
	2 months	2.3 ± 0.5	2.3 ± 0.6	2.3 ± 0.5	0.850
	EOT	0.8 ± 0.3	0.7 ± 0.2	0.8 ± 0.3	0.614
	P-value	< 0.001* < 0.001 **	< 0.001* < 0.001 **	< 0.001* < 0.001 **	
**Abdominal ultrasound findings in TB peritonitis cases (*n*** **= 12)**					
Amount of ascites					
Baseline(*n* = 12)	Marked	6(50.0)	2(33.3)	4(66.7)	0.567
	Moderate	6(50.0)	4(66.7)	2(33.3)	
2 months(*n* = 12)	Marked	2(16.7)	0	2(33.3)	0.164
	Moderate	3(25.0)	1(16.7)	2(33.3)	
	Mild	7(58.3)	5(83.3)	2(33.3)	
6 months(*n* = 12)	Moderate	2(16.7)	0	2(33.3)	
	Mild	10(83.3)	6(100.0)	4(66.7)	
EOT(*n* = 12)	No ascites	12(100.0)	6(100.0)	6(100.0)	-
**Amount of ascites compared to baseline**					
2 months(*n* = 12)	Regression	9(75.0)	5(83.3)	4(66.7)	0.505
	Stationary	3(25.0)	1(16.7)	2(33.3)	
6 months(*n* = 12)	Regression	12(100)	6(100.0)	6(100)	-
	Resolution	0	0	0	
EOT(*n* = 12)	Resolution	12(100.0)	6(100.0)	6(100.0)	-
**Computed tomography (CT) findings in cases with TB pleurisy (*n*** **= 21)**					
**Amount of pleural effusion**					
Baseline	Marked	5(23.8)	2(18.2)	3(30.0)	0.754
	Moderate	13(61.9)	7(63.6)	6(60.0)	
	Mild	3(14.3)	2(18.2)	1(10.0)	
2 months	Moderate	3(14.3)	1(9.1)	2(20.0)	0.709
	Mild	15(71.4)	8(72.7)	7(70.0)	
	No effusion	3(14.3)	2(18.2)	1(10.0)	
6months	Mild	4(19.0)	0	4(40.0)	**0.020**
	No effusion	17(81.0)	11(100.0)	6(60.0)	
EOT	No effusion	21(100.0)	11(100.0)	10(100.0)	-
**Amount of pleural effusion compared to baseline**					
2 months	Regression	18(85.7)	9(81.8)	9(90.0)	0.593
	Resolution	3(14.3)	2(18.2)	1(10.0)	
6 months	Regression	4(19.0)	0	4(40.0)	**0.020**
	Resolution	17(81.0)	11(100.0)	6(60.0)	
EOT	Resolution	21(100.0)	11(100.0)	10(100.0)	-

*P-value between Baseline and 2 months

**P-value between Baseline and EOT

## Discussion

This study revealed a high prevalence of vitamin D deficiency among newly diagnosed patients with EPTB. TB cases showed significantly lower vitamin D levels compared to their age and sex-matched healthy controls. Our findings are consistent with earlier studies demonstrated that active pulmonary TB patients had lower serum vitamin D levels compared to their household contacts and healthy controls [5, 20, 21]. Also, Hammami et al. [34] reported that vitamin D levels were significantly lower among cases of EPTB in comparison with controls and vitamin D deficiency was an independent predictor of EPTB. Similarly, Balgi. et al. [35] found lower vitamin D concentrations in both extrapulmonary and pulmonary TB cases with patients with TB meningitis had significantly lower levels of vitamin D in comparison with other forms of TB. Another study by Pareek et al. [36] showed that the patients with EPTB had lower mean vitamin D (25-OH D) concentration as compared with pulmonary TB and doubling in serum vitamin D concentration significantly reduced the risk of EPTB.

This association between vitamin D deficiency and pulmonary or extrapulmonary TB could be explained by the immunomodulatory role for vitamin D. It plays a crucial role in regulating both adaptive and innate host immune defense against TB. Vitamin D enhances the phagocytic capacity and granuloma formation of monocytes and macrophages, and increases the production of antimicrobial peptides such as cathelicidin, leading to killing of intracellular mycobacterium tuberculosis [15].

Low serum 25(OH)D3 concentrations in EPTB patients in our study may be attributed to several mechanisms such as inadequate dietary intake as reflected by the high frequency of low BMI and higher rates of unemployment among the TB patients indicating lower socioeconomic status. Moreover, 80.9% of TB patients were living in urban areas. Increasing urbanization has been associated with tendency to spend most time indoors, leading to insufficient exposure to sunlight. Additionally, the TB disease itself might have led to restricted physical activity, lack of adequate exposure to sunlight and consequent low concentrations of vitamin D. Another hypotheses that could explain vitamin D deficiency in TB cases is that vitamin D behaves as a negative acute phase reactant in TB, as has been documented in other conditions such as surgery [37] and during immune restoration syndrome in TB-HIV co-infection [38].In addition, Vitamin D level is estimated by measuring serum 25(OH)D3 concentrations. The 25(OH)D3level mainly reflects protein-bound vitamin D and may not represent the level of free active form of vitamin D. Consequently, during inflammation, similarly to levels of several other blood acute-phase proteins, the concentration of albumin decreases and might modify 25(OH)D3level results [39].

Baseline laboratory parameters in our study showed significantly lower albumin levels and hemoglobin concentrations in TB patients as compared with healthy controls. Low serum albumin levels is attributed to severe malnutrition associated with TB as well as albumin is a negative acute phase protein, so its concentration decreases in the context of significant inflammation and infection as in TB [40]. Several previous studies have demonstrated that anemia is highly prevalent in active TB cases and can be attributed to iron deficiency secondary to TB associated malnutrition and iron depletion [41].

In phase II of the study, we investigated the effect of vitamin D supplementation when added to the first line anti-TB drugs on response to treatment parameters including clinical improvement, laboratory, and radiological parameters in patients with EPTB.

An overall clinical improvement was noted in all EPTB patients, with significant weight gain during and after anti-TB treatment. The administration of vitamin D3 supplementation did not affect the overall treatment duration, except in the subgroup of patients with TB pleurisy who had shorter treatment duration.

Patients in vitamin D3 supplementation group had a greater weight gain during and at the end of TB treatment, compared to the group who received only anti-TB drugs.

Several studies have indicated that weight gain is associated with successful treatment outcomes in patients with pulmonary and extrapulmonary TB [42–44]. Conversely, lesser weight gain or weight loss were associated with poor outcomes [42, 43], or relapse [45]. In a recent study [44] evaluating treatment response in extrapulmonary tuberculosis in a low-resource setting, in TB cases with successful treatment outcomes, ≥ 5% weight gain was noted in 73 and 83% of the cases at 2 months and at treatment completion.

Furthermore, patients in vitamin D3 supplementation group showed significant improvements in hemoglobin concentration, serum albumin levels, as well as lower inflammatory markers; ESR and CRP level, both at 2 months and at the end of TB treatment. Radiological assessment demonstrated positive outcomes with vitamin D3 supplementation, particularly in patients with TB pleurisy who had a higher rate of full resolution of pleural fluid after 6 months of anti-TB treatment. Also, among TB peritonitis cases in vitamin D3 supplementation group, a higher percentage of patients experienced mild ascites at both 2 months and 6 months, though this was not statistically significant.

Compared to our results, studies investigated the effect of vitamin D supplementation on the treatment course of active pulmonary TB demonstrated variable results.

In a study by Nursyam et al. [22], pulmonary TB patients given a daily dose of vitamin D at 10,000 IU (250 mcg) for 6 weeks had significantly higher sputum conversion rates and radiological improvement compared to the placebo group. Similarly, Kota et al. [23] reported that in type 2 diabetes patients with pulmonary TB receiving 60,000 IU of vitamin D3 supplementation per week alongside anti-TB treatment, the duration of sputum conversion to 100% negative for AFB was 6 weeks compared to 8 weeks in the control group.

Salahuddin et al. [24] reported greater weight gain and accelerated radiological recovery in pulmonary TB patients who received two doses of 600,000 IU vitamin D3 administered intramuscularly, in comparison to the placebo group. Also, Hassanein., et al. [20] demonstrated that administration of single dose of 200,000IU vitamin D3 during pulmonary TB treatment has resulted in a rapid decline in sputum conversion time compared to the group receiving standard anti-TB treatment alone. Sato and colleagues [21] reported that in cured patients with active pulmonary TB, serum vitamin D levels showed a significantly negative correlation with time duration until sputum conversion, as well as platelets count and CRP and a significant positive correlation with serum albumin. They stated that these correlations reflect the anti-inflammatory effect of vitamin D and suggest that a low serum vitamin D level may not only be a risk factor for the development of active TB but it may also be related to poor treatment outcomes in active TB patients [21].

However, Martineau et al. [25] reported that the administration of vitamin D3 supplementation in four fortnightly doses of 2.5 mg (100,000 IU) had no effect on time to sputum culture conversion. However, vitamin D supplementation accelerated normalization of ESR and serum CRP and reduced chemokine production [26]. Additionally, two randomized-controlled clinical trials conducted by Wejse et al. [27] and Ganmaa et al. [28] showed that after administration of three doses of 100,000IU vitamin D2 given at 0, 5 and 8 months and four biweekly doses of 3.5 mg (140,000 IU) vitamin D3 respectively, no significant difference in the rate of mortality and sputum clearance was recognized in comparison with placebo group. Also, Ganmaa et al. [28] reported that no significant effect of vitamin D supplementation was seen on serum concentrations of the acute-phase reactants CRP, albumin, or ESR.

Our study reported normal serum calcium levels and no significant difference before and after vitamin D3 supplementation. This finding aligns with earlier studies that explored higher doses of vitamin D supplementation in the treatment course of TB with no reported hypercalcemia [22, 46, 47]. Studies have shown that there is a wide margin of safety, as serum 25(OH)-vitamin D concentrations > 400 ng/ml appear to be necessary before hypercalcemia develops [48, 49]. Compared to our results, Hassanein et al. [20] found a statistical significant difference between serum calcium at the start of anti-TB treatment and that measured at the end of 2nd month after initiation of vitamin D therapy, with elevated serum calcium level detected in 36.7% of patients. However, this was asymptomatic in all cases.

To the best of our knowledge, this is the first study to evaluate the role of vitamin D3 supplementation in the treatment course of patients with EPTB (including patients with TB lymphadenitis, TB pleurisy and TB peritonitis). However, the study has some limitations, being a single centre study and the small sample size limit the generalizability of the results. Therefore, studies with larger sample sizes that recruit patients with other forms of EPTB are still needed.

## Conclusions

This study showed high prevalence of vitamin D deficiency among newly diagnosed patients with EPTB, compared to healthy controls. Additionally, vitamin D3 supplementation has been beneficial in treatment of EPTB, and associated with higher weight gain, as well as better improvement of serum concentrations of hemoglobin and acute-phase reactants. Therefore, we recommend assessing vitamin D level in TB patients starting anti-TB therapy, followed by administration of vitamin D supplementation according to the level of vitamin D insufficiency/deficiency detected.

## Data Availability

The datasets used and analysed during the current study available from the corresponding author on reasonable request.

## Abbreviations

EPTB: extrapulmonary tuberculosis
TB: Tuberculosis
WHO: World health organization
AFB: acid-fast bacilli
MDR-TB: multi-drug resistant TB
BMI: body mass index
CRP: C reactive protein
